# Bioinformatic curation and alignment of genotyped hepatitis B virus (HBV) sequence data from the GenBank public database

**DOI:** 10.1186/s40064-016-3312-0

**Published:** 2016-10-28

**Authors:** Trevor G. Bell, Mukhlid Yousif, Anna Kramvis

**Affiliations:** Hepatitis Virus Diversity Research Unit, Department of Internal Medicine, University of the Witwatersrand, 7 York Road, Parktown, South Africa

**Keywords:** Bioinformatics, Blast, Genbank, Hepatitis B virus, HBV, Sequence alignment

## Abstract

**Background:**

Hepatitis B virus (HBV) DNA sequence data from thousands of samples are present in the public sequence databases. No publicly available, up-to-date, multiple sequence alignments, containing full-length and subgenomic fragments per genotype, are available. Such alignments are useful in many analysis applications, including data-mining and phylogenetic analyses.

**Results:**

By issuing a query, all HBV sequence data from the GenBank public database was downloaded (67,893 sequences). Full-length and subgenomic sequences, which were genotyped by the submitters (30,852 sequences), were placed into a multiple sequence alignment, for each genotype (genotype A: 5868 sequences, B: 4630, C: 7820, D: 8300, E: 2043, F: 985, G: 189, H: 108, I: 23), according to the results of offline BLAST searches against a custom reference library of full-length sequences. Further curation was performed to improve the alignment.

**Conclusions:**

The algorithm described in this paper generates, for each of the nine HBV genotypes, multiple sequence alignments, which contain full-length and subgenomic fragments. The alignments can be updated as new sequences become available in the online public sequence databases. The alignments are available at http://hvdr.bioinf.wits.ac.za/alignments.

## Background

### Public sequence databases

Direct (Sanger) DNA sequencing (Sanger et al. 1977) is a relatively inexpensive and routine procedure in molecular biology. DNA sequence data are used to identify or compare organisms, genes or mutations, or to confirm laboratory procedures. Comparing sequence data from one or more samples to each other and to a known “reference” alignment is a standard procedure. Evolutionary relationships between samples are elucidated by phylogenetic analyses, which typically involve DNA sequences from many organisms of interest, and from many related samples for comparison.

In 1979, the Los Alamos Sequence Database was established by Walter Goad (Kanehisa et al. 1984). With support from the National Institutes of Health, this database was expanded in 1982 and renamed “GenBank” (Bilofsky et al. 1986). Since 1982, the number of nucleotides in GenBank doubles approximately every 18 months (Benson et al. 2015).

The International Nucleotide Sequence Database Collaboration (INSDC) (Karsch-Mizrachi et al. 2012) is a collection of three publicly available nucleotide (DNA or RNA) sequence databases, which synchronize data daily. The collection consists of the DNA DataBank of Japan (DDBJ, located in Japan), the European Molecular Biology Laboratory (EMBL, located in the United Kingdom) and GenBank (located in the United States of America). The latest release of the database (Release 210.0, from October 15, 2015)^1^ contains 188372017 loci and 202237081559 bases, from 188372017 sequences, totaling approximately 742 gigabytes.

Researchers share sequence data with the academic community by submitting sequences to these public databases. This is generally a requirement when sequences are included in a publication. In addition to nucleotide sequence data, various optional meta-data fields are included with sequence submissions. These fields include the name of the organism from which the sequence data originated, the names of the authors or researchers, a draft publication title, co-ordinates of any coding regions within the sequence, and the date of the submission. If coding regions are specified, the resulting protein translation is stored in the database automatically. Although sequence data submitted to the databases is checked for integrity, no checks are performed on text submitted into the free-form text fields, such as the “Author” or “Note” fields.

Data are submitted and extracted from the database in a flat, plain text (“ASCII”) file format. Sequence data are typically extracted from the database by issuing a query to the database via the database web-site. Results are displayed on the web-site and can be downloaded in various file formats. As data have been submitted over many years, during which time, sequencing and computing techniques have evolved and improved, the reliability and accuracy of these data can vary. Thus, unavoidable artefacts, errors and inconsistencies may be present in the data.

### Hepatitis B virus

Hepatitis B virus (HBV), which is globally distributed, is a member of the family *Hepadnaviridae*, a group of hepatotropic viruses found in humans, various other primates, rodents and some bird species. Chronic infection in humans may cause severe liver damage, including cirrhosis and hepatocellular carcinoma (liver cancer).

Although the HBV genome, which is circular and approximately 3200 nucleotides in length, is partially double-stranded DNA, it replicates via a single-stranded RNA intermediate (Summers et al. 1978). The HBV genome codes for seven proteins, in four overlapping, open reading frames (ORFs) (Michel and Tiollais 1987).

Nucleotide variation between HBV strains is common, as proof-reading during replication is absent in HBV (Steinhauer and Holland 1986). Sequences, which diverge by more than 7.5 % across the full genome, are considered to be different genotypes (Kramvis et al. 2008). These genotypes can differ in length ranging from 3182 to 3248 nucleotides because of the presence of indels (insertions or deletions) of 3–36 nucleotides.

Sequence divergence calculations and phylogenetic analyses have resulted in the identification of nine genotypes (“strains”) of HBV, distributed geographically (Norder et al. 2004; Kramvis et al. 2005; Yu et al. 2010; Kramvis 2014).

In 2008, sequence analysis of the complete genome of a single isolate (AB231908) from Vietnam found it to be closely related to three previously described “aberrant” Vietnamese strains (Hannoun et al. 2000) and a ninth genotype, I, was proposed (Tran et al. 2008). This proposal was not accepted, because the mean genetic divergence of these four strains from genotype C was 7 % and the recombination analysis was not robust (Kurbanov et al. 2008). With the availability of additional sequences (Arankalle et al. 2010; Osiowy et al. 2010; Yu et al. 2010), the nucleotide divergence of most of these sequences relative to genotype C was at least 7.5 %, with good bootstrap support for the group, thus meeting the criteria for genotype assignment (Kramvis et al. 2008).

Contemporary co-ordinate numbering of the HBV genome assigns a position of 1 to the first “T” nucleotide after the *Eco*R1 restriction site ($\hbox {G}|\hbox {AA}{} \mathbf T \hbox {TC}$) when sequenced in the forward direction. Prior to this convention, the start of the Core region (position 1901 in the contemporary system) was considered to be the first position of the HBV genome. The HBV genome is circular and position 1 happens to occur within the full S (surface) ORF. Therefore, the full nucleotide sequence of the S gene straddles position 1, and the full S ORF does not run contiguously within a linear sequence in the standard orientation. Thus, the start of the S ORF varies depending on the genotype, being 2854 for genotype A, 2848 for genotypes B to F and H, and 2884 for genotype G. In all genotypes, the S ORF ends at position 835. Extracting the full S gene sequence from linear sequence data therefore requires additional sequence processing.

Although several websites and databases related to HBV sequence data exist (Table 1), the scope and focus of these vary widely, with some intended for medical or clinical diagnostic use only. Additionally, sets of full-length reference sequences for subgenotypes A (Cai et al. 2016) and C (Zhu et al. 2015) are available.

The aim of the present study is to provide updated, curated, alignments of each HBV genotype (A to I), consisting of all available full-length and subgenomic fragments from the GenBank public database, where the genotype could be mined from the GenBank submission. Data from these alignments can be used as reference data sets and in other downstream analyses.

## Methods

**Fig. 1:**
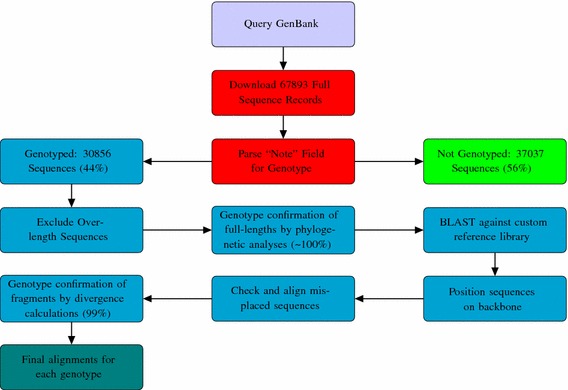
The workflow used to prepare the data set and alignments of each genotype of HBV

An overview of the methodology used is shown in Fig. 1.

### GenBank download

Full sequence records were downloaded as plain text files from GenBank using the following query:

*(hepatitis b virus[organism] not rna[title] not clone[title] not clonal[title] not patent[title] not recombinant[title] not recombination[title] and 200:99,999[sequence length])*

This query excluded sequences shorter than 200 nucleotides in length, which is the current lower limit imposed by GenBank. Additionally, sequences in which the title contained the words specified in the query above were excluded. The organism was limited to “hepatitis B virus”. The plain text file therefore contained full sequence records of both HBV “full-length” sequences and subgenomic fragments. The file was approximately 220 megabytes in size and contained 67,893 sequences.

### Genotype parsing

Computer programs to process the sequence data text file, were written in the Python programming language (van Rossum 1995) running on a GNU/Linux computer. Components of the BioPython library (Cock et al. 2009) were used in some of the programs.

HBV genotype is recorded, by some submitters only, in the free-form “note” text field of the sequence record, using different notations or formats. Examples include “genotype: A1”, “genotype: C”, “subtype: ayw2; genotype: D” or “subtype: A1; genotype: A”. A Python script was written to extract the genotype from the “note” field, which is part of the “sequence features” section of the downloaded full sequence records. The case (upper or lower) of the text and punctuation were ignored. If this field contained the text “type” (indicating the possible presence of the words “genotype”, “subgenotype” or “subtype”), the script considered the presence of a single letter from “A” to “I”, optionally followed by one or more digits (0–9), to be the genotype of the sample. These (“genotyped”) samples constituted the data set used in this study. The remaining samples (those in which the “note” field was empty, or for which the contents of the “note” field could not be interpreted) were excluded from the remainder of the study. If the genotype was specified in another field, this was also ignored, which may be a limitation.

Sequences consisting of exactly the typical number of nucleotides, for each genotype, were classified as “Complete” full-length sequences. Sequences consisting of fewer than the typical number of nucleotides, for each genotype, were classified as “Subgenomic” and were at least 200 nucleotides in length. Sequences exceeding the typical number of nucleotides (overlength sequences), for each genotype, were excluded from the study.

Neighbour-joining phylogenetic analyses (*not shown and available on request*) were undertaken on all full-length samples in the data set (which did not contain wobbles^2^ or “N” characters, and which contained the sequence motif “ATG” at position 155). With the exception of five sequences, these analyses confirmed that the genotype, which the submitters included in the GenBank record, was correct, and that this genotype had been correctly extracted from the full sequence record. Phylogenetic analyses were performed using the neighbor-joining method with 1000 bootstrap replicates via the Molecular Evolutionary Genetic Analysis (MEGA) software program (version 5) (Tamura et al. 2011), with the Kimura two-parameter distance estimation. Sequences were considered to be the same genotype if they shared the same node on the tree and their sequence divergence was $\le$7.5 %.

**Fig. 2:**
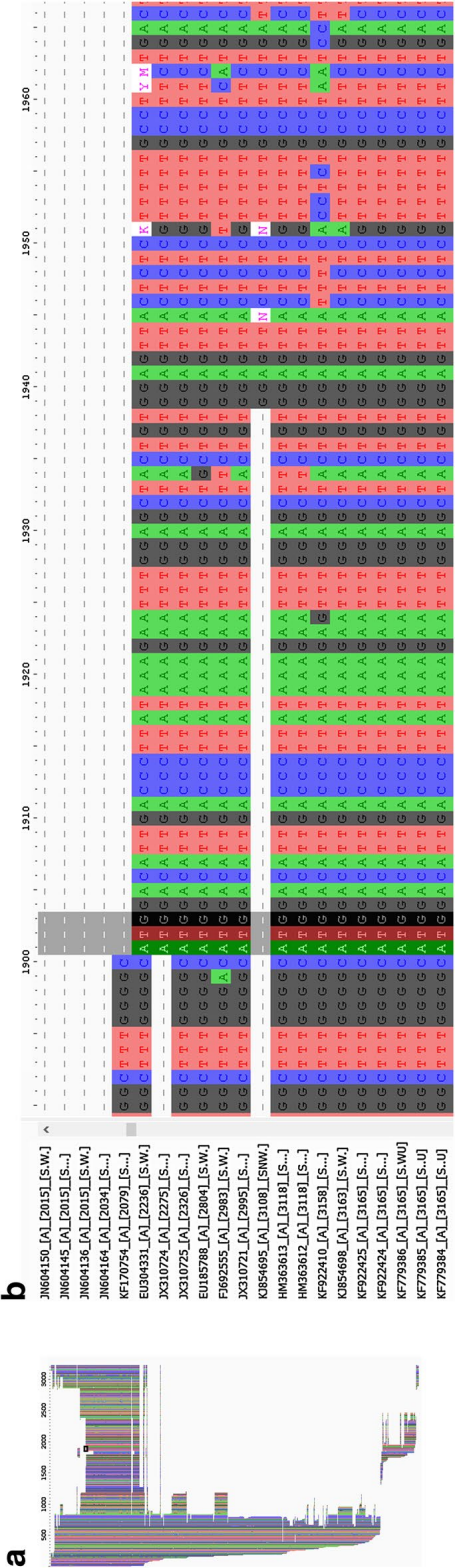
Representative final alignment. **a** A screenshot of the entire alignment of genotype A, as visualized by the *AliView* program (Larsson 2014); **b** a zoomed view of the rectangular region from **a**. The annotated FASTA ID of the sequences are to the left of the sequence data. The start of the Core region (nucleotides ATG) at position 1901 is highlighted for reference

**Table 1:** Currently available HBV websites and databases

Name	url	Usage	References
Geno2Pheno[hbv]	www.genafor.org/g2p_hbv/index.php	Drug resistance mutations, escape mutant analsysis	Beerenwinkel et al. (2003)
HBV Blast Search	http://www.bioafrica.net/blast/hbvblast.html	Genotyping, drug resistance database	Oliveira et al. (2005)
HBV STAR	http://www.vgb.ucl.ac.uk/starn.shtml	Genotyping	Myers et al. (2006)
HBVRegDB	http://lancelot.otago.ac.nz/HBVRegDB/	Annotation, alignments, information about conserved regions	Panjaworayan et al. (2007)
HBVdb	http://hbvdb.ibcp.fr/	Genotyping, annotation, drug resistance database	Hayer et al. (2013)
HBVseq	http://hivdb.stanford.edu/HBV/HBVseq/development/HBVseq.html		
Hepatitis Virus Database	http://s2as02.genes.nig.ac.jp/	Genotyping, sequence alignment and map viewing	
Hepatitis Virus Database	http://www.ibibiobase.com/projects/hepatitis/index.htm		
NCBI Genotyping Tool	http://www.ncbi.nlm.nih.gov/projects/genotyping/	Genotyping	Rozanov et al. (2004)
Oxford HBV Subtyping Tool	http://www.bioafrica.net/rega-genotype/html/subtypinghbv.html	Genotyping	Oliveira et al. (2005)
RegaDB	http://regaweb.med.kuleuven.be/software/regadb		Libin et al. (2007)
SeqHepB	http://www.seqhepb.com/	Sequence analysis, genotyping, detection of clinically important mutations	Yuen et al. (2007)

**Table 2:** Extraction of data and preparation of data set

	Genotyped	Overlength (removed)	Raw	No BLAST hits (removed)	Placed	Consensus with gaps (removed)	Mismatch (included)	Overhang (removed)	Final
A	5959	14	5945	0	5945	77	0	0	5868
B	4734	24	4710	0	4710	79	0	1	4630
C	8205	72	8133	90	8043	222	0	1	7820
D	8508	103	8405	6	8399	99	0	0	8300
E	2053	2	2051	0	2051	8	0	0	2043
F	1009	4	1005	1	1004	19	0	0	985
G	250	2	248	4	244	55	0	0	189
H	113	3	110	0	110	2	0	0	108
I	25	1	24	0	24	1	0	0	23
Total	30,856	225	30,631	101	30,530	562	0	2	29,966

**Table 3:** Classification of sequences in the final alignment

Genotype	C..*	CN.*	C.W*	CNW*	S..*	SN.*	S.W*	SNW*	Total	C**U	S**U
A	424	9	87	3	4676	64	596	9	5868	0	149
B	458	5	119	2	2957	60	986	43	4630	7	8
C	868	4	210	5	5684	92	916	41	7820	2	130
D	544	1	127	6	6276	61	1257	28	8300	35	163
E	165	0	41	0	1594	8	234	1	2043	0	0
F	104	2	37	2	722	49	63	6	985	2	5
G	17	0	1	0	157	0	14	0	189	0	4
H	11	0	5	0	76	0	16	0	108	0	0
I	5	0	0	0	14	0	4	0	23	0	0
Total	2596	21	627	18	22,156	334	4086	128	29,966	46	459

“C” in the first position represents “Complete” sequences, “S” in the first position represents “Subgenomic” sequences, “N” in the second position indicates at least one “N” character in the sequence, otherwise “.”; “W” in the third position indicates at least one wobble in the sequence, otherwise “.”, “U” in the last position indicates an “Unverified” sequence, otherwise “.”, “*” indicates that either value for that position is included whereas "**" indicates either value for two adjoining columns, the “Total” column is the total of the first eight columns

### Genotype alignments

The data set consisted of 30,856 genotyped sequences, of which 4214 were full-length sequences. Multiple sequence alignment software programs cannot (reliably) align data sets consisting of thousands of sequences, of such varying lengths, covering an entire genome. Thus, an alignment containing both full-length and subgenomic fragments (correctly placed), for each genotype, was created as follows. Initial placement of sequences, for each genotype in turn, was performed by the BLAST (“Basic Local Alignment Search Tool”) program (Altschul et al. 1990). BLAST, which is available as an online service or as an offline, stand-alone program, compares sequences (typically a query against a reference library) of nucleotides or amino acids, and returns a series of alignment matches. A reference library, for each genotype, was prepared by selecting the first three full-length sequences of each genotype (in the order in which they were provided by GenBank), which did not contain wobbles or “N” characters, and with the nucleotide sequence “ATG” (the START motif) at genome position 155. Using the first three sequences in the file was sufficient and gave good results. However, this can easily be modified because the source code will be made available in due course. Thereafter, each sequence for a given genotype was submitted to the “blastn” implementation of the BLAST program (version 2.2.28+, running on a GNU/Linux computer). The BLAST results were interrogated and the co-ordinates of the first match was used as the position at which the query sequence should be placed. However, if the the length of a BLAST match was shorter than the length of the query sequence, the second match was also used. This was necessary, as linear full S open reading frame subgenomic fragments straddle position 1 of the genome, which is circular. The BLAST algorithm yields matches for the regions of the S ORF upstream and downstream of position 1. As the genotype of each query sequence was known, a BLAST library of three reference sequences, of the same genotype, was sufficient. To prevent the creation of multiple small gaps in the alignments, the gap-opening and gap-extension parameters of “blastn” were each set to a value of 5.

An alignment, for each genotype, was then constructed by placing all full-length sequences (for each genotype in turn) into a new alignment. Subgenomic fragments were then added to this alignment by placing each fragment (at its BLAST co-ordinate) onto an empty template,^3^ consisting only of gap (“-”) characters of the appropriate genotype length. Genotype A, for example, is 3221 nucleotides in length. Each subgenomic fragment of genotype A, was then positioned onto its own template (initially consisting of 3221 gaps), starting at the corresponding co-ordinate according to each BLAST result. This sequence was then added to the alignment, and the process was repeated for the next subgenomic fragment. The entire process was repeated using data for each genotype in turn, to create a separate alignment for each genotype.

Inspecting the alignments by eye in the *AliView* alignment viewer (Larsson 2014), which can “zoom out” to display hundreds of sequences at a time, showed that some subgenomic fragments were placed incorrectly by one position. In most cases, these sequences started one position downstream of the correct position in the sequence. These discrepancies may be explained by the variation in the HBV genome, the length and position of the subgenomic fragment, the nature of the BLAST algorithm, and the composition of the reference library. These misplaced sequences were processed as follows. The number of mismatches between each sub-genomic fragment (as positioned in the alignment) and a consensus sequence of that alignment was determined. Fragments containing more than 8 % mismatches were selected for checking. The cut-off of 8 % was determined by testing a range of values and selecting the one at which the number of excluded sequenced plateaued *(not shown)*. Each selected (query) fragment was then pairwise aligned with the consensus sequence, using the Needleman–Wunsch algorithm (Needleman and Wunsch 1970), as implemented by the EMBOSS “needle” program (Rice et al. 2000). If the consensus sequence in the pairwise alignment contained at least one gap character (indicating the presence of an insertion in the query sequence), then the query sequence was discarded from the genotype alignment. For the remaining pairwise alignments, the number of mismatches was recalculated. Fragments, which now contained 8 % or fewer mismatches, were retained in the alignment, and those containing more than 8 % mismatches were discarded. This approach requires that only selected sequences are pairwise aligned. No multiple sequence alignment is required. The master alignment itself is constructed by positioning fragments according to BLAST search results.

A small number of subgenomic fragments extended beyond the length of the genotype in some of the genotype alignments. These “overhang” sequences, which were correctly placed and did not exceed the mismatch threshold of 8 %, contained a small number of insertions and were removed from the alignment.

Additional information about each sequence was appended to the FASTA ID for reference. Examples of the modified FASTA ID are as follows:

$$\begin{aligned} {\texttt {AA123456\_[A]\_[0522]\_[SNWU]}}\\ {\texttt {ZZ987654\_[C]\_[3215]\_[C...]}} \end{aligned}$$

The characters preceeding the first underscore (“_”) are the GenBank accession number. The single, uppercase letter, within square brackets following this, is the genotype of the sequence, as recorded by the submitters, and in most cases confirmed in this study. Next in square brackets is the sequence length in the alignment. The final set of four letters in square brackets provides details about various sequence features, as follows. The first letter is a “C” if the sequence is a “Complete” full-length, or an “S” if the sequence is a subgenomic fragment. If the sequence contains at least one “N” character, the next letter is an “N”, otherwise it is a single period (“.”). If the sequence contains at least one wobble nucleotide, the next letter is a “W”, otherwise it is a single period. The final of the four letters is a “U” if the sequence has been flagged by GenBank as “unverified”, otherwise it is a single period.

In order to confirm the submitter’s genotype assignment of subgenomic fragments, sequence divergence calculations were performed. Nucleotide divergence calculations and phylogenetic analyses are the two primary methods used for genotyping. Undertaking phylogenetic analyses of subgenomic fragements of varying lengths at different positions across the genome is not feasible. Therefore, an in-house nucleotide divergence tool (Bell and Kramvis 2016) was used to calculate the nucleotide divergence of the subgenomic fragments, compared to a reference set containing all genotypes, covering the same genomic region. It was found that the genotype extracted, according to the submitter, was correct in 99 % of the sequences (*not shown*).

## Results and discussion

The alignment for genotype A is shown in Fig. 2. The outcomes of the alignent and curation processes are detailed in Tables 2 and 3. Table 2 shows the data extracted and the preparation of the alignment. Table 3 depicts the classification of the sequences in the final alignment.

Using the search query reported in the Methods section above,^4^ 67,893 full sequence records were downloaded on 29 November 2015. A genotype was recorded by the submitters in 30,856 (44 %) of these sequences. The word “recombinant” or “recombination” occurred in the “note” field 168 times, and all 168 of these sequences were excluded from the study.

GenBank requires that two subgenomic fragments, sequenced from the same sample, be submitted as a single “full-length” entry, with many consecutive “N” characters placed between the two subgenomic fragments. Following a GenBank query, these sequences with the “N” padding are returned as full-length sequences and not as two separate subgenomic fragments. Such sequences should not be used in phylogenetic analyses or as reference sequences, as they are not complete. In the present algorithm, the “N” characters in such sequences are removed and replaced with gaps. The resulting sequence is therefore appropriately no longer considered as a “full-length” sequence, and the FASTA ID for these sequences is tagged with an “S” (“Subgenomic”) character.

A knowledge of the genotypes circulating in a community and the prevalence of certain mutations can assist in deciding on better management and treatment options. Comparative analysis of sequences can also trace transmission routes and aid in design of preventative measures. Globally and locally the different genotypes can have distinct geographic distributions (Kramvis et al. 2005; Kramvis 2014). Moreover, the genotype of HBV can influence the clinical outcome of HBV infection because it can affect the frequency of HBeAg-positivity, the age at which HBeAg loss occurs, and thus the mode of transmission (Kramvis, *in press*). Therefore, the natural history of HBV infection varies in the different regions of the world. The genotypes also differ in their propensity to develop mutations that can influence the course of disease. Phylogenetic analyses, mutation reporting and molecular characterization of HBV full genome and subgenomic sequences are therefore important downstream applications that can utilize the curated alignments generated in the present study.

These alignments, which include all genotyped full-length and sub-genomic fragments, correctly placed, are as comprehensive and as accurate as possible. The algorithm in this study unavoidably misclassified five sequences (AB367800, AB367801, AB367802, AB367803, AB367804) because the terms used in the note field (“... patient with type B chronic hepatitis...”) suggest that this sample may belong to HBV genotype B (or that it is HBV, rather than hepatitis A virus or hepatitis C virus), whereas our analysis shows that it belongs to HBV genotype C. This couldf be avoided by the inclusion of a “genotype” field in the GenBank record.

These alignments are intended to be used for further downstream analyses. However, in order to meet the specific needs of a given project, some additional curating of the alignments may be necessary, such as removing sequences, which are not required. The modified FASTA ID annotations provided, include the genotype, the sequence length and additional tags, and can be used to further filter the sequences, as required. If only full-length sequences are required, sub-genomic sequences can be selected and deleted from the alignments using a multiple sequence alignment viewer/editor, such as MEGA6 (Tamura et al. 2013), GeneDoc (Nicholas et al. 1997) or Aliview (Larsson 2014). Full-length sequences, or sub-genomic sequences covering a region of interest, can be used as outgroups or as sister sequences in phylogenetic analyses. The alignments, for each genotype, can be downloaded at the following url: http://hvdr.bioinf.wits.ac.za/alignments. These alignments may be used freely by the academic research community, with the *proviso* that this paper is acknowledged and cited.

Sequence data for specific regions of the genome only, can be obtained by submitting an alignment to the Babylon Tool (Bell and Kramvis 2015), which extracts (and optionally translates) nucleotides from one or more ORFs into separate FASTA files. For example, an alignment containing only nucleotides covering the S ORF for genotype A can be downloaded by submitting the genotype A alignment to the Babylon Tool and selecting the S ORF (position 155 to position 835). Any sequences, which do not cover the entire S ORF, can be deleted from the alignment in a multiple sequence alignment editor.

The Mutation Reporter Tool (Bell and Kramvis 2013) summarizes nucleotide or amino acid distribution at specified loci of interest. The tool can, optionally, include or exclude sequences based on a FASTA ID regular expression. This can be used to report on the nucleotide distribution of only the full-length (“Complete”) sequences, for example. The “include” regular expression used in this example would be “${\texttt {\_}{\mathtt {\backslash }}{\mathtt {[C}}{\mathtt {[{}^\wedge ]}}{\mathtt {]}}}$”, that is, an underscore, followed by an open square bracket and a “C” character, not followed by a close square bracket. The last component is particularly required, if the input alignment contains genotype C sequences, to prevent matching with the genotype annotation, “[C]”. Note that the closing square bracket, within the regular expression list, is not escaped with a backslash character.

Phylogenetic analyses of complete genomes of HBV is the gold standard for accurate genotype classification. By selecting representative sequences from the various genotype alignments, the genotype of a newly sequenced strain can be determined. It is also possible to genotype using the complete S region. However, this method will not preclude the presence of recombination.

GenBank is a valuable database for accessing nucleotide sequences of HBV. The method developed in the present study, provides sequence alignnments, of both complete and subgenomic fragments of the HBV genome, which have been downloaded from GenBank. The genotype of these sequences has been confirmed and the sequences aligned and curated. Therefore, these prepared alignments are a useful resource for comparative analyses of genotype distributions, transmission routes and development of mutations.

## Availability, usage and future work

At present, preparation of the alignments is not automated to run in an unattended fashion. Future work includes automating the process of downloading sequence data from GenBank and running the various programs and scripts. We undertake to release the source code of the programs and scripts via the source-code hosting website *GitHub* within one year of the date of publication of this article.

## Footnotes

1
ftp://ftp.ncbi.nih.gov/genbank/gbrel.txt.

2 Ambiguous or degenerate bases.

3 Backbone/scaffold/skeleton.

4 (Hepatitis b virus[organism] not rna[title] not clone[title] not clonal[title] not patent[title] not recombinant[title] not recombination[title] and 200:99,999[sequence length]).

## References

[CR1] Altschul SF, Gish W, Miller W, Myers EW, Lipman DJ (1990). Basic local alignment search tool. J Mol Biol.

[CR2] Arankalle VA, Gandhe SS, Borkakoty BJ, Walimbe AM, Biswas D, Mahanta J (2010). A novel HBV recombinant (genotype I) similar to Vietnam/Laos in a primitive tribe in eastern India. J Viral Hepat.

[CR3] Beerenwinkel N, Däumer M, Oette M, Korn K, Hoffmann D, Kaiser R, Lengauer T, Selbig J, Walter H (2003). Geno2pheno: estimating phenotypic drug resistance from HIV-1 genotypes. Nucl Acids Res.

[CR4] Bell TG, Kramvis A (2013) Mutation reporter tool: an online tool to interrogate loci of interest, with its utility demonstrated using hepatitis B virus. Virol J. doi:10.1186/1743-422X-10-62 10.1186/1743-422X-10-62 PMC3749809 23433201

[CR5] Bell TG, Kramvis A (2015). Bioinformatics tools for small genomes, such as hepatitis B virus. Viruses.

[CR6] Bell TG, Kramvis A (2016) The study of hepatitis B virus using bioinformatics. In: Abdurakhmonov I (ed) btitleBioinformatics—updated features and applications, 1st edn. InTechOpen, Rijeka. http://bit.ly/BioinformaticsChapterHBV

[CR7] Benson DA, Clark K, Karsch-Mizrachi I, Lipman DJ, Ostell J, Sayers EW (2015) GenBank. Nucl Acids Res 43(Database issue):30–35 10.1093/nar/gku1216 PMC4383990 25414350

[CR8] Bilofsky HS, Burks C, Fickett JW, Goad WB, Lewitter FI, Rindone WP, Swindell CD, Tung CS (1986). The GenBank genetic sequence databank. Nucl Acids Res.

[CR9] Cai Q, Zhu H, Zhang Y, Li X, Zhang Z(2016) Hepatitis B virus genotype A: design of reference sequences for sub-genotypes. Virus Genes. doi:10.1007/s11262-016-1307-0 10.1007/s11262-016-1307-0 27002608

[CR10] Cock PJ, Antao T, Chang JT, Chapman BA, Cox CJ, Dalke A, Friedberg I, Hamelryck T, Kauff F, Wilczynski B, de Hoon MJ (2009). Biopython: freely available Python tools for computational molecular biology and bioinformatics. Bioinformatics.

[CR11] de Oliveira T, Deforche K, Cassol S, Salminen M, Paraskevis D, Seebregts C, Snoeck J, van Rensburg EJ, Wensing AM, van de Vijver DA, Boucher CA, Camacho R, Vandamme AM (2005). An automated genotyping system for analysis of HIV-1 and other microbial sequences. Bioinformatics.

[CR12] Hannoun C, Norder H, Lindh M (2000). An aberrant genotype revealed in recombinant hepatitis B virus strains from Vietnam. J Gen Virol.

[CR13] Hayer J, Jadeau F, Deleage G, Kay A, Zoulim F, Combet C (2013). HBVdb: a knowledge database for hepatitis B virus. Nucl Acids Res.

[CR14] Kanehisa M, Fickett JW, Goad WB (1984). A relational database system for the maintenance and verification of the Los Alamos sequence library. Nucl Acids Res.

[CR15] Karsch-Mizrachi I, Nakamura Y, Cochrane G, Miyano S, Nakamura H, Sugano S, Danchin A, Savakis B, Weissenbach J, Weng Z, Salzberg S (2012). The international nucleotide sequence database collaboration. Nucl Acids Res.

[CR16] Kramvis A (2014). Genotypes and genetic variability of hepatitis B virus. Intervirology.

[CR17] Kramvis A, Kew M, François G (2005). Hepatitis B virus genotypes. Vaccine.

[CR18] Kramvis A, Arakawa K, Yu MC, Nogueira R, Stram DO, Kew MC (2008). Relationship of serological subtype, basic core promoter and precore mutations to genotypes/subgenotypes of hepatitis B virus. J Med Virol.

[CR19] Kurbanov F, Tanaka Y, Kramvis A, Simmonds P, Mizokami M (2008). When should “I” consider a new hepatitis B virus genotype?. J Virol.

[CR20] Larsson A (2014) Aliview: a fast and lightweight alignment viewer and editor for large datasets. Bioinformatics 30(22):3276–3278. doi:10.1093/bioinformatics/btu531. http://bioinformatics.oxfordjournals.org/content/30/22/3276.full.pdf+html 10.1093/bioinformatics/btu531 PMC4221126 25095880

[CR21] Libin P, Deforche K, Laethem K, Camacho R, Vandamme A(2007) Regadb: an open source, community-driven hiv data and analysis management environment. In: btitleFifth European HIV Drug Resistance Workshop, Cascais, Portugal

[CR22] Michel M-L, Tiollais P (1987). Structure and expression of the hepatitis b virus genome. Hepatology.

[CR23] Myers R, Clark C, Khan A, Kellam P, Tedder R (2006). Genotyping hepatitis B virus from whole- and sub-genomic fragments using position-specific scoring matrices in HBV STAR. J Gen Virol.

[CR24] Needleman SB, Wunsch CD (1970). A general method applicable to the search for similarities in the amino acid sequence of two proteins. J Mol Biol.

[CR25] Nicholas KB, Nicholas HB, Deerfield DW (1997). GeneDoc: analysis and visualization of genetic variation. Embnew News.

[CR26] Norder H, Courouce AM, Coursaget P, Echevarria JM, Lee SD, Mushahwar IK, Robertson BH, Locarnini S, Magnius LO (2004). Genetic diversity of hepatitis B virus strains derived worldwide: genotypes, subgenotypes, and HBsAg subtypes. Intervirology.

[CR27] Osiowy C, Kaita K, Solar K, Mendoza K (2010). Molecular characterization of hepatitis B virus and a 9-year clinical profile in a patient infected with genotype I. J Med Virol.

[CR28] Panjaworayan N, Roessner SK, Firth AE, Brown CM (2007). HBVRegDB: annotation, comparison, detection and visualization of regulatory elements in hepatitis B virus sequences. Virol J.

[CR29] Rice P, Longden I, Bleasby A (2000). EMBOSS: the European Molecular Biology Open Software Suite (2000). Trends Genet.

[CR30] Rozanov M, Plikat U, Chappey C, Kochergin A, Tatusova T (2004). A web-based genotyping resource for viral sequences. Nucl Acids Res.

[CR31] Sanger F, Nicklen S, Coulson AR (1977). DNA sequencing with chain-terminating inhibitors. Proc Natl Acad Sci USA.

[CR32] Steinhauer DA, Holland JJ (1986) Direct method for quantitation of extreme polymerase error frequencies at selected single base sites in viral RNA. J Virol 57:219–228. http://jvi.asm.org/content/57/1/219.full.pdf+html 10.1128/jvi.57.1.219-228.1986 PMC252718 3001347

[CR33] Summers J, Smolec JM, Snyder R (1978). A virus similar to human hepatitis B virus associated with hepatitis and hepatoma in woodchucks. Proc Natl Acad Sci USA.

[CR34] Tamura K, Peterson D, Peterson N, Stecher G, Nei M, Kumar S (2011). MEGA5: molecular evolutionary genetics analysis using maximum likelihood, evolutionary distance, and maximum parsimony methods. Mol Biol Evol.

[CR35] Tamura K, Stecher G, Peterson D, Filipski A, Kumar S (2013). MEGA6: Molecular Evolutionary Genetics Analysis version 6.0. Mol Biol Evol.

[CR36] Tran TTH, Trinh TN, Abe K (2008). New complex recombinant genotype of hepatitis B virus identified in Vietnam. J Virol.

[CR37] van Rossum G (1995) Python tutorial, Technical Report CS-R9536. Centrum voor Wiskunde en Informatica (CWI), Amsterdam

[CR38] Yu H, Yuan Q, Ge S-X, Wang H-Y, Zhang Y-L, Chen Q-R, Zhang J, Chen P-J, Xia N-S (2010). Molecular and phylogenetic analyses suggest an additional hepatitis B virus genotype “I”. PLOS One.

[CR39] Yuen LK, Ayres A, Littlejohn M, Colledge D, Edgely A, Maskill WJ, Locarnini SA, Bartholomeusz A (2007). SeqHepB: a sequence analysis program and relational database system for chronic hepatitis B. Antivir Res.

[CR40] Zhu HL, Wang CT, Xia JB, Li X, Zhang ZH (2015). Establishment of reference sequences of hepatitis B virus genotype C subgenotypes. Genet Mol Res.

